# The Molecular Mechanism of Amyloid β42 Peptide Toxicity: The Role of Sphingosine Kinase-1 and Mitochondrial Sirtuins

**DOI:** 10.1371/journal.pone.0137193

**Published:** 2015-09-03

**Authors:** Magdalena Cieślik, Grzegorz A. Czapski, Joanna B. Strosznajder

**Affiliations:** Department of Cellular Signalling, Mossakowski Medical Research Centre Polish Academy of Sciences, Warsaw, Poland; Indian Institute of Toxicology Reserach, INDIA

## Abstract

Our study focused on the relationship between amyloid β 1–42 (Aβ), sphingosine kinases (SphKs) and mitochondrial sirtuins in regulating cell fate. SphK1 is a key enzyme involved in maintaining sphingolipid rheostat in the brain. Deregulation of the sphingolipid metabolism may play a crucial role in the pathogenesis of Alzheimer’s disease (AD). Mitochondrial function and mitochondrial deacetylases, i.e. sirtuins (Sirt3,-4,-5), are also important for cell viability. In this study, we evaluated the interaction between Aβ_1–42,_ SphKs and Sirts in cell survival/death, and we examined several compounds to indicate possible target(s) for a strategy protecting against cytotoxicity of Aβ_1–42_. PC12 cells were subjected to Aβ_1–42_ oligomers and SphK inhibitor SKI II for 24–96 h. Our data indicated that Aβ_1–42_ enhanced SphK1 expression and activity after 24 h, but down-regulated them after 96 h and had no effect on Sphk2. Aβ_1–42_ and SKI II induced free radical formation, disturbed the balance between pro- and anti-apoptotic proteins and evoked cell death. Simultaneously, up-regulation of anti-oxidative enzymes catalase and superoxide dismutase 2 was observed. Moreover, the total protein level of glycogen synthase kinase-3β was decreased. Aβ_1–42_ significantly increased the level of mitochondrial proteins: apoptosis-inducing factor AIF and Sirt3, -4, -5. By using several pharmacologically active compounds we showed that p53 protein plays a significant role at very early stages of Aβ_1–42_ toxicity. However, during prolonged exposure to Aβ_1–42_, the activation of caspases, MEK/ERK, and alterations in mitochondrial permeability transition pores were additional factors leading to cell death. Moreover, SphK product, sphingosine-1-phosphate (S1P), and Sirt activators and antioxidants, resveratrol and quercetin, significantly enhanced viability of cells subjected to Aβ_1–42_. Our data indicated that p53 protein and inhibition of SphKs may be early key events responsible for cell death evoked by Aβ_1–42_. We suggest that activation of S1P-dependent signalling and Sirts may offer a promising cytoprotective strategy.

## Introduction

Alzheimer’s disease (AD) is a major cause of age-related cognitive dysfunction and dementia. According to the amyloid cascade hypothesis, an alteration of amyloid β (Aβ) production and metabolism is recognised as the first pathologically important event which is responsible for activation of a molecular cascade that leads to synaptic dysfunction and neurodegeneration [1]. Aβ peptides are a major component of extracellular protein aggregates, known as senile plaques, which are the main neuropathological hallmark of AD. However, recent discoveries have shown that Aβ in aggregated form is biologically non-active and that the most toxic forms of Aβ are oligomers of variable molecular weight [2, 3]. It was demonstrated that the level of Aβ oligomers is increased in AD brains and correlates with disease severity [4]. Also, *in vivo* experiments confirmed the pronounced role of oligomerisation in Aβ toxicity. Lesne et al. [2], by using transgenic Tg2576 mice which express the human Aβ precursor protein (APP), demonstrated that the appearance of Aβ oligomers in the brain correlates with memory decline in this animal AD model. Moreover, they demonstrated that intracerebral injection of Aβ oligomers into healthy rats causes memory deficits.

Among the many processes impaired in the AD brain, deregulation of the sphingosine biostat seems to be especially important and may be responsible for this disease’s pathogenesis and pathomechanism. Sphingosine kinases (SphK1 and SphK2) are responsible for the biosynthesis of sphingosine-1-phosphate (S1P). These enzymes are crucial for the sphingolipid biostat between S1P and ceramide and for cell survival and death. Intracellular S1P, synthesised by SphK1, exerts pro-survival effects, influences calcium mobilisation, gene expression, cell growth and proliferation. S1P may act as a second messenger, but it can also be transported to the extracellular space and may affect cell function *via* stimulation of five G-protein coupled receptors (S1P1–5). The S1P pool, synthesised by membrane-bound SphK1, seems to be pro-survival, whereas S1P produced by nuclear/cytosolic SphK2 may activate pro-apoptotic pathways. Within the cell, S1P is dephosphorylated by S1P phosphatase or hydrolysed by S1P lyase (SPL). The balance between ceramide, sphingosine and S1P controls cell proliferation, migration and viability [5, 6].

The growing body of evidence indicates the great importance of deregulation of the sphingolipid metabolism in AD [7]. Ceramide was elevated in AD brains and peaked at a very early stage of the disease, and the level of sphingolipids, such as ceramide, dihydrosphingosine and phytosphingosine, in the plasma has been proposed as a potential biomarker of AD [8, 9]. Both alterations of sphingosine metabolism enzymes and changes at the levels of sphingosine, S1P, and ceramide were observed in AD patients compared to age-matched healthy controls [10]. SphK activity declines in the brains of AD patients, which is followed by a decreasing level of S1P [6]. Lowered expression of SphK1 and increased expression of SPL were also reported in *post-mortem* samples of AD brain tissue [11]. These alterations correlated with Aβ deposits and the Braak stage of the disease. However, opposite results were published by Takasugi et al. [12], who demonstrated upregulation of SphK2 activity in the brains of AD patients and indicated its role in the activation of BACE1.

Also, experimental studies *in vitro* and *in vivo* can confirm the important role of sphingolipid metabolism alterations in the pathomechanism of AD [13–17]. Deregulation of the sphingolipid biostat leads to the accumulation of ceramide, whose role in AD was described by Yuyama et al. [18], and its involvement in the molecular mechanism of neuronal cell death mediated by poly(ADP-ribose)polymerase-1 (PARP1) was demonstrated by Czubowicz and Strosznajder [19]. However, the role of Aβ_1–42_ in the modulation of sphingolipid kinases and sirtuins has not been fully elucidated. Recent studies indicate that altering the activity of silent information regulator 2 proteins, also known as sirtuins, might affect AD pathology. Sirtuins are a family of highly conserved NAD-dependent deacetylases. Some of them also exert ADP ribosylation activity [20–22] or, as Sirt5, demalonylation and desuccinylation activity [23]. Sirt3 may control cellular [NADP]/[NADPH] ratios and may serve as a sensor of the metabolic state of cells and the ROS defence system. There are seven members of the sirtuin family, i.e. Sirt1-Sirt7, which regulate stress response, DNA repair and apoptosis [24–28]. A recent study focused on mitochondria-located Sirt3, -4, -5, and particularly on Sirt3 [29]. These sirtuins deacetylate and activate mitochondrial enzymes that are involved in the metabolism of amino acids and fatty acids. Moreover, Sirt3 modulates the function of electron transport chain proteins and anti-oxidative defences. Sirt3 prevents apoptosis by modulation of mitochondrial transition pore proteins and the level of free radicals [29, 30]. According to the most recent findings, activators of Sirt1 and inhibitors of Sirt2 would exert a beneficial effect in AD [24]. It has been demonstrated that Sirt1 attenuates amyloidogenic processing of APP in cell culture studies *in vitro* and in transgenic mouse models of AD. Sirt1 increases α-secretase activity and non-amyloidogenic cleavage of APP. In consequence, APP processing is shifted towards reducing pathological, toxic Aβ liberation by β- and γ-secretases [31]. Furthermore, activation of α-secretase (ADAM10) by Sirt1 also induces the Notch signalling pathway, which is known to repair neuronal damage in the brain [32].

Despite the enormous amount of research that has been conducted so far, there is no treatment for AD. The aim of this study was to focus on the relationship between Aβ_1–42_, SphK1 and mitochondrial Sirts and on the molecular events engaged in apoptotic signalling in order to identify target(s) for improving the therapeutic strategy.

## Materials and Methods

### Chemicals

The following antibodies were used in the study: anti-phospho-Gsk-3β(Ser9) (Cell Signaling Technology, Beverly, MA, USA), anti-Gsk-3β and anti-AIF (Santa Cruz Biotechnology, Dallas, TX, USA), anti-rabbit IgG, anti-GAPDH, and anti-PARP1 (Sigma-Aldrich, St. Louis, MO, USA), and anti-mouse IgG (GE Health Care UK, Little Chalfont, Buckinghamshire, UK). HFIP-treated Amyloid β1–42 and Amyloid β scrambled were obtained from rPeptide (rPeptide, Bogart, GA, USA). Protease inhibitor cocktail Complete was obtained from Roche Diagnostics GmbH (Mannheim, Germany). Omega(7-nitro-2–1,3-benzoxadiazol-4-yl)(2S,3R,4E)-2-aminooctadec-4-ene-1,3-diol (NBD-sphingosine) was obtained from Avanti Polar Lipids Inc. (Alabaster, AL, USA). Reagents for reverse transcription (High Capacity RNA-to-cDNA Master Mix) and quantitative PCR (Taqman Assays and Gene Expression Master Mix) were obtained from Applied Biosystems (Foster City, CA, USA). Serum-free Neurobasal-A medium and supplement B27 were from Invitrogen (Carlsbad, CA). Dulbecco’s Modified Eagle’s Medium (DMEM), fetal bovine serum (FBS), horse serum (HS), penicillin, streptomycin, glutamine, 3-(4,5-dimethyl-2-tiazolilo)-2,5-diphenyl-2H-tetrazolium bromide (MTT), TRI-reagent, DNase I, DTT, polyethyleneimine (PEI), anhydrous DMSO, and all other reagents were obtained from Sigma-Aldrich (St. Louis, MO, USA).

### Preparation of Aβ oligomers

**Fig 1:**
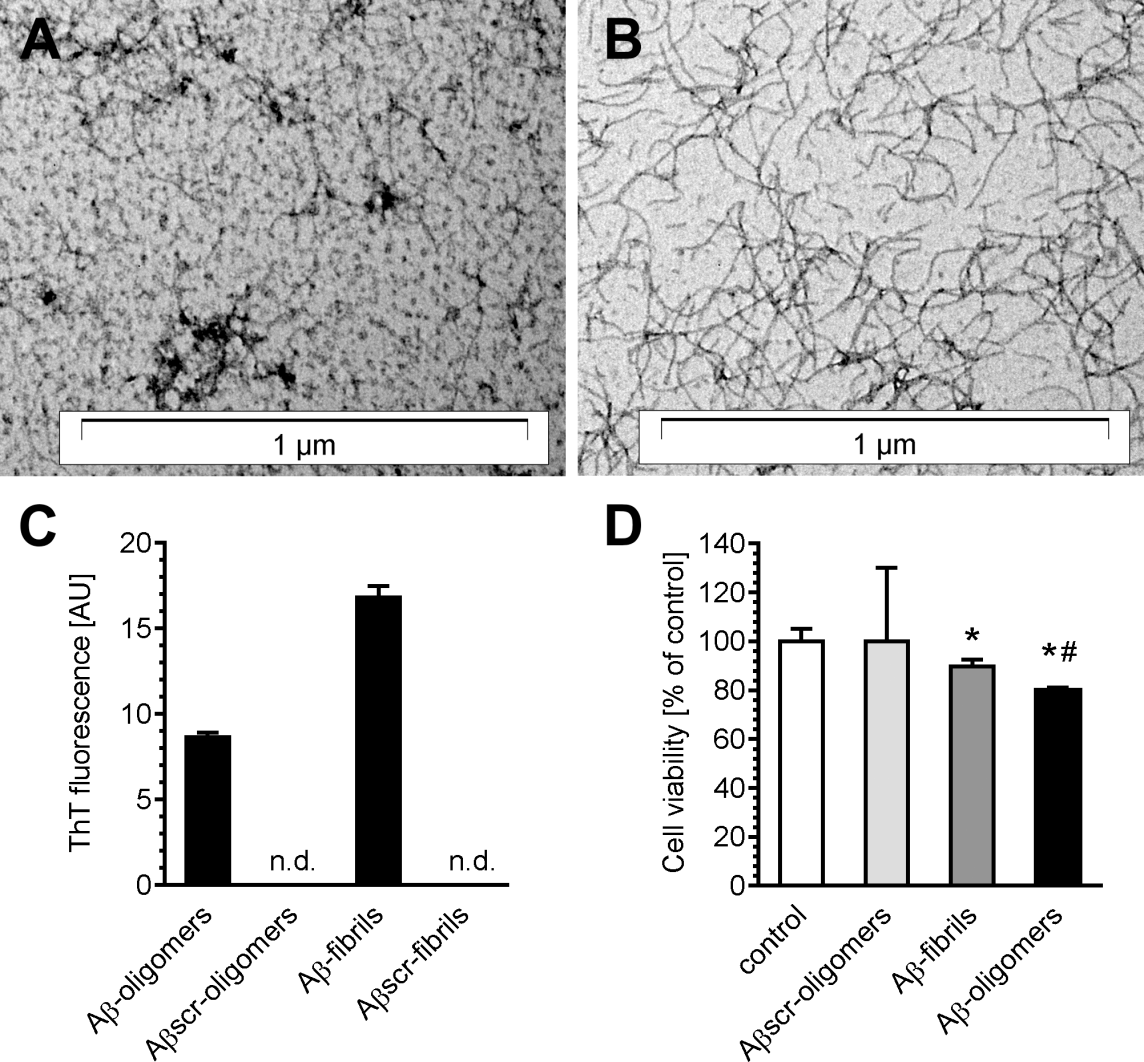
The effect of Aβ on PC12 cells viability. (A) Representative electron-microscopic image of oligomeric form of Aβ; (B) representative electron-microscopic image of fibrillar form of Aβ; (C) analysis of Thioflavin T (ThT) fluorescence in various Aβ preparations, n.d.—non-detected; (D) the effect of oligomeric and fibrillar Aβ_1–42_ or scrambled Aβ at 1 μM concentration on PC12 cell viability after 24-h treatment; *p < 0.05, comparing to control group; #*p* < 0.05, comparing to Amyloid β fibrils, by one-way ANOVA followed by Newman-Keuls test.

Oligomerization of Aβ1–42 was performed according to Stine et al. [33]. Amyloid β was dissolved (5 mM) in anhydrous DMSO and further diluted in ice-cold cell culture medium (Phenol Red-free Ham’s F-12) to 100 μM final concentration. After 30 s vortexing, Aβ solution was incubated at 4°C for 24 h. In accordance with previous data, 24 h incubation of Aβ_1–42_ monomers at physiological ionic strength and neutral pH at 4°C yielded small-size oligomeric assemblies of Aβ, whereas incubation at low ionic strength and acidic pH at 37°C produced mainly long fibrils (Fig 1A and 1B). In addition, conformation state of Aβ was confirmed using Thioflavin T (ThT), which is a benzothiazole dye binding to amyloid fibrils. Incubation of Aβ preparations with ThT evoked a rise in the fluorescence of ThT, indicating the presence of Aβ aggregates (Fig 1C). The cytotoxicity of Aβ_1–42_ oligomers (AβO) and fibrils was confirmed on PC12 cells after 24-h incubation. In addition, Aβ_1–42_ with scrambled sequence (Aβscr) was used as a negative control (Fig 1D).

### Cell culture

Rat pheochromocytoma PC12 cells were kind gift from Prof. A. Eckert (University of Basel, Basel, Switzerland) [34, 35]. The cells were cultured in DMEM supplemented with 10% heat-inactivated fetal bovine serum, 5% heat-inactivated horse serum, 2 mM L-glutamine, 50 U/ml penicillin, 50 μg/ml streptomycin in 5% CO_2_ atmosphere at 37°C.

### Cell treatment protocols

To avoid binding of Aβ by serum albumins, all experiments were performed in serum-free Neurobasal-A medium supplemented with B27 supplement. Equal PC12 cell numbers were seeded into dishes or 96-well 0.1% PEI-coated plate, and after 24 h they were treated for 24–96 h with freshly prepared oligomeric Aβ (1 μM) or with sphingosine kinases inhibitor SKI II (10 μM, dissolved in DMSO), p53 inhibitor α-pifithrin (20 μM, dissolved in DMSO), pan-caspase inhibitor Z-VAD-FMK (40 μM, dissolved in DMSO), mitochondrial permeability transition pore blocker cyclosporine A (2 μM, dissolved in ethanol), MEK/ERK inhibitor U0126 (1 μM, dissolved in DMSO), inhibitor of protein kinase C GF109203X (1 μM, dissolved in DMSO), resveratrol (25 μM, dissolved in DMSO), quercetin (100 μM, dissolved in DMSO/water 1:1), sphingosine-1-phosphate (1 μM, dissolved in 0.4% BSA in water), fingolimod-P (100 pM, dissolved in chloroform), PARP inhibitors 3-aminobenzamide (5 mM, dissolved in DMSO/water 1:1), and PJ34 (20 μM, dissolved in water). Appropriate solvent was added to respective controls.

### Determination of cell survival using MTT test

Cellular viability was evaluated by the reduction of 2-(4,5-dimethylthiazol-2-yl)-2,5-diphenyltetrazolium bromide (MTT) to formazan. After the treatment with investigated compounds, MTT (0.25 mg/ml) was added and cells were incubated for 2 h. The medium was removed, the cells were lysed with DMSO, and the absorbance at 595 nm was recorded.

### Determination of free radicals using 2',7'-dichlorofluorescein (DCF)

The level of reactive oxygen species (ROS) in PC12 cells was assessed using a fluorogenic probe—2',7'-dichlorodihydrofluorescein diacetate (H_2_DCF-DA). After the incubation in the presence of tested compounds, medium was changed to pre-warmed Phenol Red-free Hanks’ buffer supplemented with 20 mM HEPES (pH 7.4) and 2.5 mM Probenecid 10 μM H_2_DCF-DA, and the incubation was continued for 50 min at 37°C. The cells were washed with pre-warmed PBS and fluorescence (λ_ex_ **=** 485 nm, λ_em_ = 525 nm) was measured in a multi-mode microplate reader FLUOstar Omega (BMG Labtech GMBH, Ortenberg, Germany).

### Determination of Sphk activity

SphK(s) activity assay was performed, as described previously [36]. After the incubation, cells were washed with iced PBS and lysed by freeze-thaw cycle in 50 mM HEPES (10 mM KCl, 15 mM MgCl_2_, 0.1% Triton X-100, 20% glycerol, 2 mM orthovanadate, 2 mM dithiothreitol, 10 mM NaF, 1 mM deoxypyridoxine, and EDTA-free protease inhibitor cocktail (pH 7.4). Lysates were cleared by centrifugation at 15,000 rpm for 5 min. The lysates and NBD-Sphingosine (10 μM final concentration) were mixed in the reaction buffer (50 mM HEPES, 15 mM MgCl_2_, and 0.5 mM KCl, 10% glycerol, and 2 mM ATP) (pH 7.4) and incubated for 30 min at 30°C. The reactions were stopped by the addition of equal amount of 1 M potassium phosphate (pH 8.5), followed by the addition of 2.5-fold chloroform/methanol (2:1) and then centrifuged at 15,000 rpm for 1 min. Only the product of reaction, NBD-sphingosine-1-phosphate (NBD-S1P), but not the substrate NBD-sphingosine, was collected in alkaline aqueous phase. After the aqueous phase was combined with an equal amount of dimethylformamide, the fluorescence value was read (λ_ex_ = 485 nm, λ_em_ = 538 nm).

### Analysis of gene expression

After the treatment with investigated compounds, cells were washed twice with ice-cold PBS, scraped from the culture dish, and centrifuged briefly (3 min, 1000 × g). RNA was isolated from the cell pellet using TRI-reagent according to the manufacturer’s protocol. Digestion of DNA contamination was performed using DNase I according to the manufacturer’s protocol. The quantity and quality of RNA were controlled via spectrophotometric analysis. Reverse transcription was performed using a High Capacity cDNA Reverse Transcription Kit according to the manufacturer’s protocol. The level of mRNA for selected genes was analysed by using TaqMan Gene Expression Assays according to the manufacturer’s instructions: ACTB-4352340E (*Actb*), Rn00591307-m1 (*Sphk1*), Rn01457923-g1 (*Sphk2*), Rn99999125_m1 (*Bcl2*l), Rn01501410-m1 (*Sirt3*), Rn01481485-m1 (*Sirt4*), Rn01450559-m1 (*Sirt5*), Rn01475306-m1 (*Aifm1*), Rn00565018-m1 (*Parp1*), Rn00560930_m1 (*Cat*), Rn00755717_m1 (*Tp53*). The other gene transcripts were analysed by using the Power SYBR Green PCR Master Mix (Applied Biosystems, Foster City, CA, USA). using primer pairs (forward/reverse): *Bax*, 5'-GACACCTGAGCTGACCTTGGA-3'/5'-GACACTCGCTCAGCTTCTTGGT-3'; *Sod2*, 5'-CGCTGGCCAAGGGAGAT-3'/5'-CCCCGCCATTGAACTTCA-3'; *Cyb5b*, 5'-TGAGCCTGACGCCCAAA-3'/5'-TGGGAACCAGTGAGAAGAAACC-3'. Plates were analysed using an ABI PRISM 7500 apparatus. The relative levels of mRNA were calculated applying the ΔΔCt method.

### Western immunoblotting

After SDS-polyacrylamide gel electrophoresis, proteins were transferred to nitrocellulose membrane, and the quality of transfer was verified with Ponceau S staining. The membranes were destained with TBS supplemented with 0.1% Tween 20 (TBST) and used for immunochemical detection in standard conditions: blocking—5% BSA in TBST for 1 h at room temperature (RT); washing—3 x TBST for 5 min at RT; primary antibody incubation—in 5% BSA at 4°C overnight; washing—3 x TBST for 5 min at RT; secondary antibody—in 5% non-fat milk in TBST for 1 h at RT; washing—3 x TBST for 5 min and 1 x in TBS for 5 min at RT. Then, chemiluminescent reaction was performed (Thermo Fisher Scientific Inc., Rockford, IL, USA). GAPDH was detected on membranes as a loading control. Densitometric analysis and size-markers based verification was performed with TotalLab4 software.

### Statistical Analysis

The results were expressed as mean values ± S.E.M. The differences between means were analysed using Student t-test or one-way analysis of variance (ANOVA) with Neuman-Keuls post-hoc test. The statistical analyses were performed by using Graph Pad Prism version 5.0 (Graph Pad Software, San Diego, CA, USA).

## Results

In the present study, we analysed the molecular network between Aβ peptide, sphingosine kinase and sirtuins in cell survival and death. Previously published data demonstrated that oligomers are the most toxic form of Aβ. To examine the various forms of Aβ in our experimental conditions, we compared the toxicity of the oligomeric and fibrilar form of Aβ_1-42_. Moreover, as a negative control we used scrambled Aβ_1-42_ (Aβ_scr_ has the same composition of amino acids but in random order), which was subjected to the same oligomerisation protocol. Our data indicated that the oligomeric form (AβO) was more toxic than the fibrils (Fig 1D), thus AβO was used in further experiments. Aβ_scr_ had no effect on cell viability, thus demonstrating that the toxic effect is specific for Aβ_1–42_.

**Fig 2:**
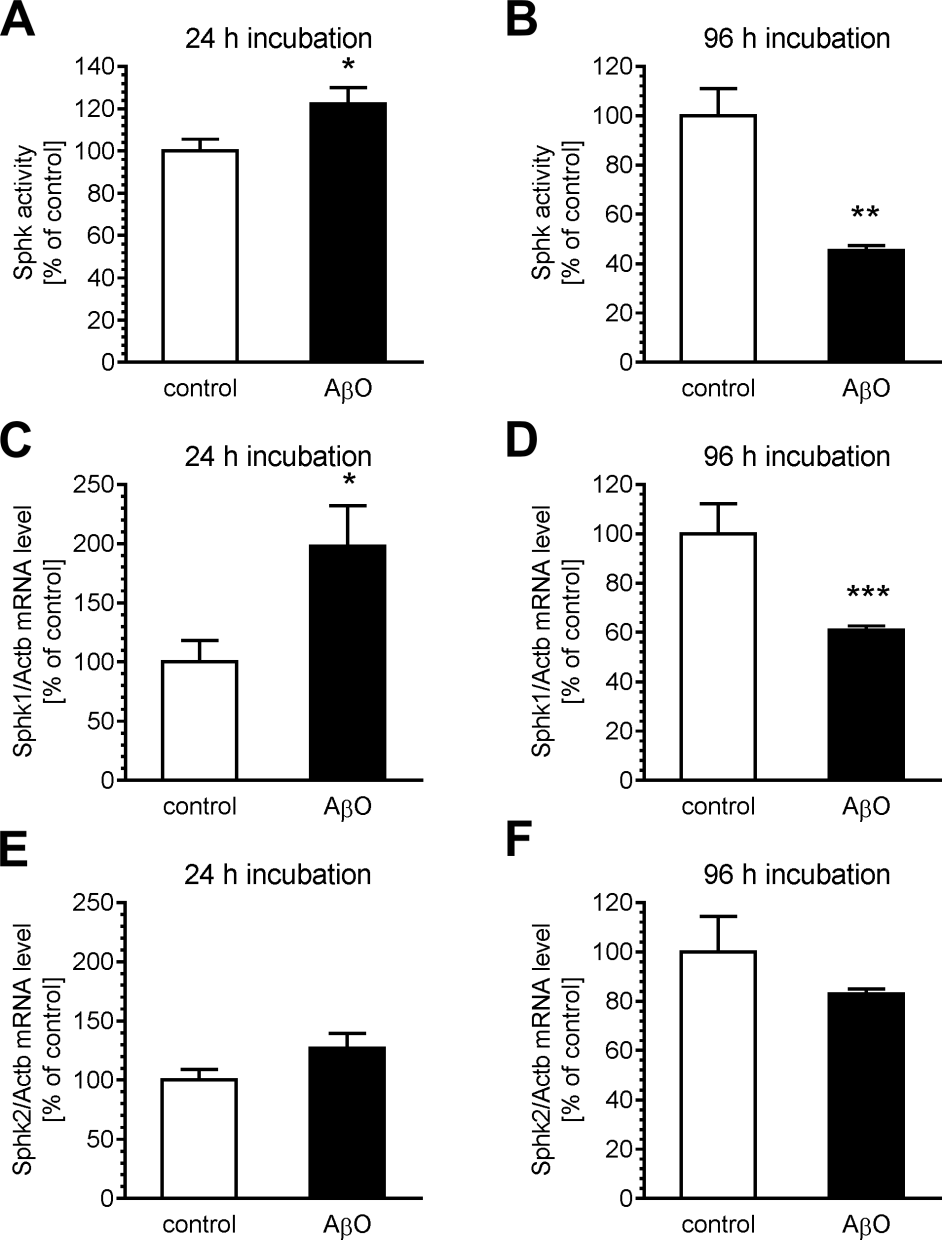
The effect of acute and prolonged treatment with AβO on expression and activity of sphingosine kinases. PC12 cells were incubated in the presence of oligomeric Aβ (AβO, 1 μM) for 24 and 96 h. (A–B) The activity of Sphk1 and Sphk2 was determined, as described in Methods. (C–F) The levels of mRNA of *Sphk1* and *Sphk2* genes were analysed by quantitative RT-PCR. The results of RT-PCR were normalized to *Actb* gene expression and are presented as the mean ± SEM from 4 independent experiments; *, **, *** for *p* < 0.05, 0.01, and 0.001, respectively, as compared with the corresponding control, by using Student’s t-test.

As is shown in Fig 2, 24 h incubation in the presence of 1 μM AβO evoked little increase in SphK activity, but after 96 h of exposure the activity of SphK was significantly reduced. It was found that the mRNA level for *Sphk1* corresponds with the activity; 24 h incubation with AβO evoked an increase, and 96 h incubation evoked a decrease in the mRNA level. However, the mRNA level for the *Sphk2* gene changed neither after 24 h incubation nor after 96 h. These data suggested that inhibition of SphK1 may be an important molecular mechanism responsible for the toxic effects of AβO.

**Fig 3:**
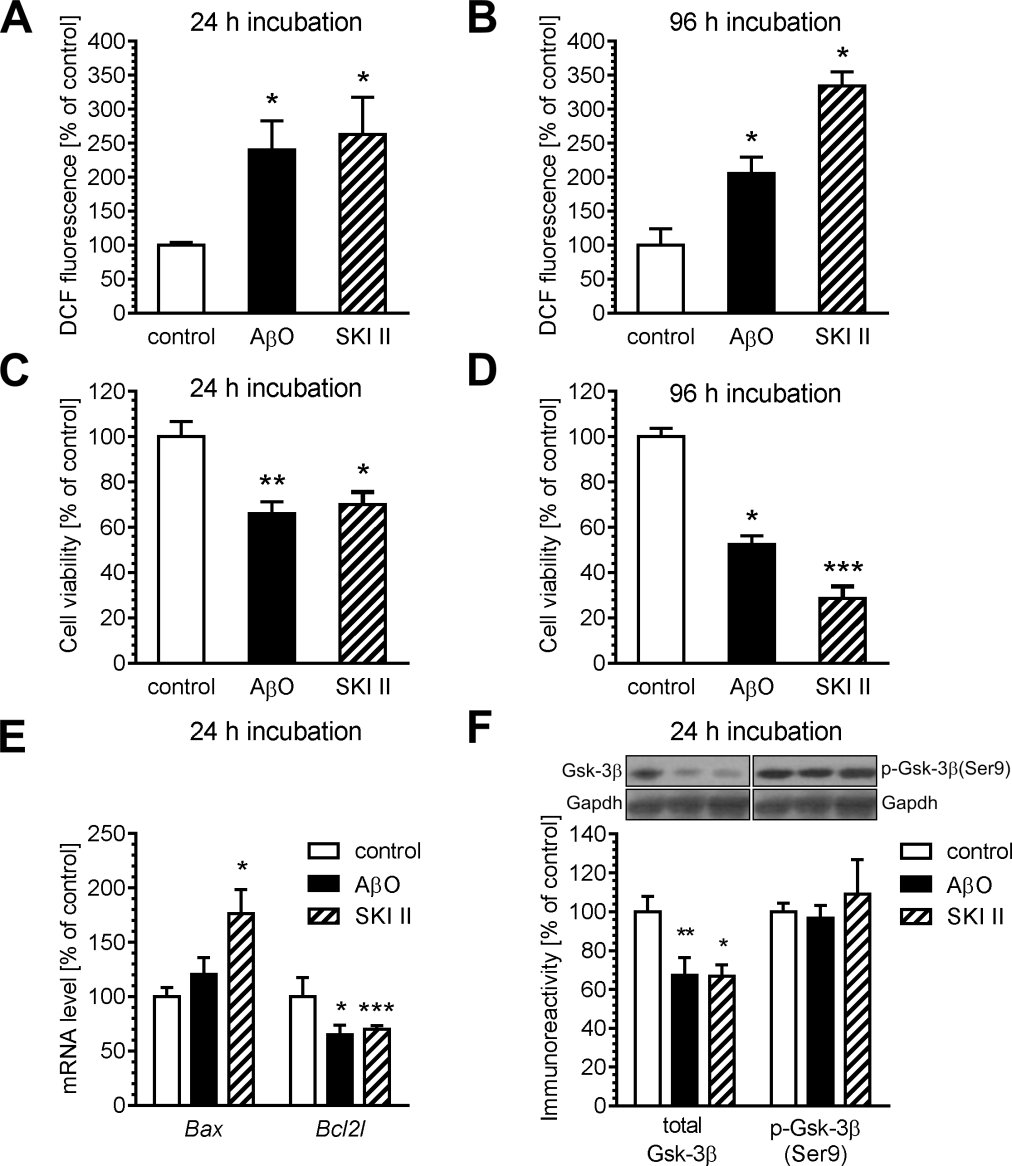
The effect of oligomeric Aβ and SKI II on oxidative stress, cell viability, level and phosphorylation of Gsk-3β, and expression of apoptosis-related genes. PC12 cells were incubated in the presence of oligomeric Aβ (AβO, 1 μM) and SKI II (10 μM) for 24 (A, C, E, F) and 96 h (B, D). (A–B) The level of oxidative stress was determined with DCF probe, as described in Methods. (C–D) Cell viability was determined by using MTT assay, as described in Methods. (E) The levels of mRNA of Bcl-2-associated X protein (Bax) and Bcl-xL (Bcl2l1) were analysed via quantitative RT-PCR. The results of RT-PCR were normalized to *Actb* gene expression. (F) The total level of Gsk-3β protein and phosphorylation at Ser9 were determined using the Western blotting method. Densitometric data were normalized to total protein level, as determined by Ponceau S staining. Data represent the mean value ± S.E.M. for 4–8 independent experiments. Gapdh is presented as a loading control. The typical pictures were presented. *p < 0.05, **p < 0.01, ***p < 0.001, as compared to the control cells, using a one-way ANOVA followed by the Newman-Keuls test.

To investigate the mechanism of AβO- and SphK-dependent toxicity, several molecular pathways were analysed by using the SphK inhibitor SKI II. The effect of AβO or SKI II on DCF fluorescence was studied to examine the possible role of free radicals (Fig 3A and 3B). DCF is a fluorogenic probe detecting free radicals and other reactive oxygen species in living cells. After 24 h incubation, AβO and SKI II evoked a similar increase in DCF fluorescence (ca. 250% of the control). The effect of prolonged 96 h incubation in the presence of SKI II was much stronger, reaching ca. 334% of an increase, as compared to a ca. 205% increase in cells incubated with AβO. The level of free radicals was negatively correlated with cell viability (Fig 3C and 3D). After 24 h incubation, AβO and SKI II evoked an equivalent decrease in cell viability by about 30–35%. The effect of prolonged 96 h incubation in the presence of SKI II was much stronger, reaching a ca. 70% decrease, as compared to a ca. 50% decrease in cells incubated with AβO. The balance between pro- and anti-apoptotic proteins after AβO and SKI II exposure was also examined. Exposing cells to AβO or SKI II resulted in up-regulation of gene expression for the pro-apoptotic Bax protein and in a decrease of the anti-apoptotic Bcl-xL protein (Fig 3E). The next goal was to analyse the possible interaction between AβO, SphK and Gsk-3β, which is an important target of Aβ-induced toxicity. Our results indicated that incubation for 24 h in the presence of AβO significantly reduced the level of the Gsk-3β protein. An inhibitor of SphK, SKI II, also reduced the level of Gsk-3β immunoreactivity after 24 h incubation. To examine whether AβO and SKI II may affect Gsk-3β function, we analysed the phosphorylation of Gsk-3β at Ser9, which is a major mechanism regulating Gsk-3β activity. Our data indicated that the level of immunoreactivity for p-Gsk-3β (Ser9) was not altered after 24 h incubation in the presence of AβO or SKI II (Fig 3F). Since the level of Gsk-3β phosphorylated on Ser9 (inactive) was not changed, our data may suggest that the level of the active form of Gsk-3β is reduced after incubation in the presence of AβO and SKI II. Experiments carried out under prolonged incubation of AβO for 96 h showed no change in the level and phosphorylation of Gsk-3β at Ser9 (S1 Fig).

**Fig 4:**
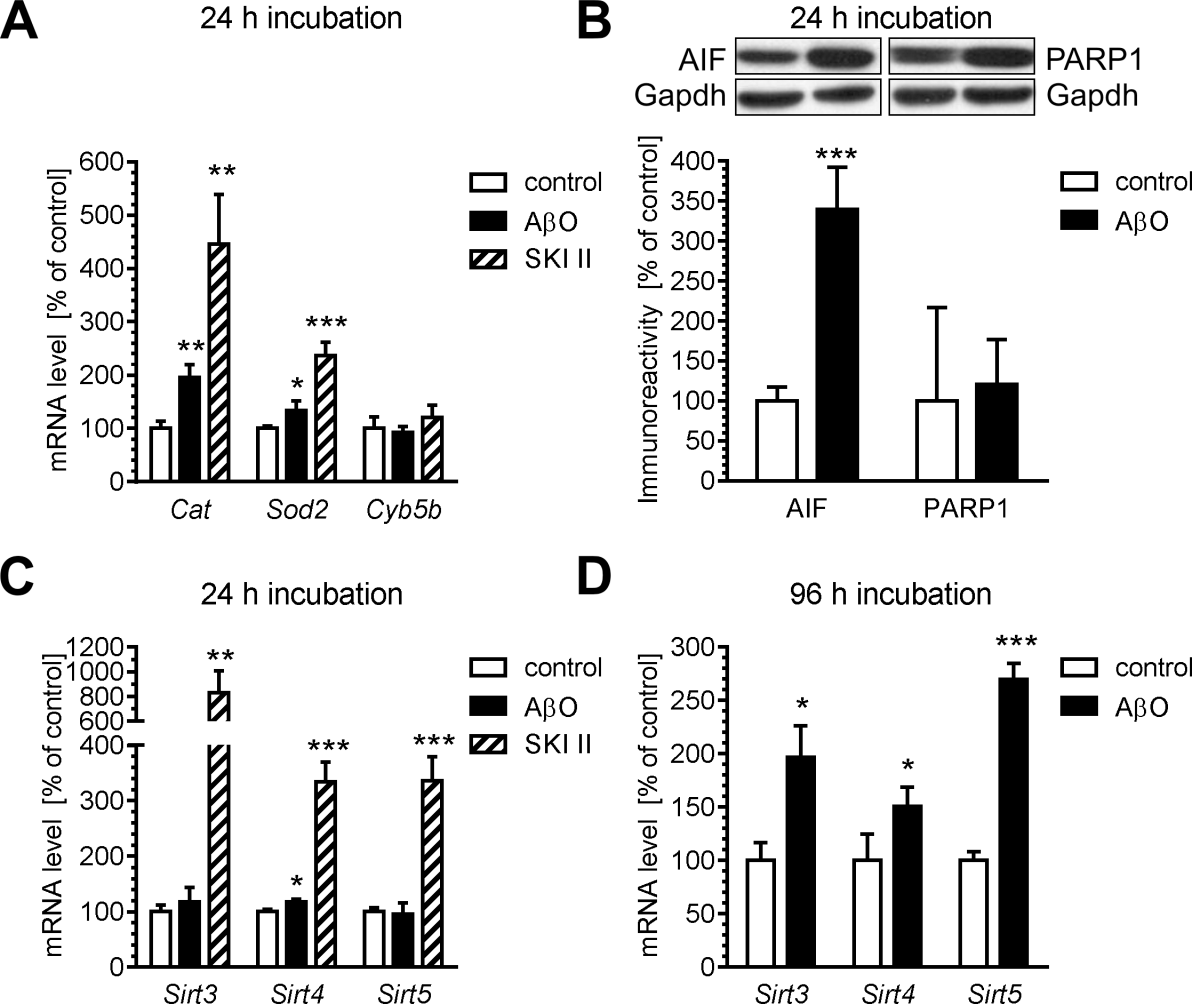
The effect of oligomeric Aβ and SKI II on mitochondrial function. PC12 cells were incubated in the presence of oligomeric Aβ (AβO, 1 μM) and SKI II (10 μM) for 24 (A–C) and 96 h (D). (A, C, D) The levels of mRNA of Catalase (*Cat*), Superoxide dismutase (*Sod*), Cytochrome b5 type B (*Cyb5b*), Sirtuin 3 (*Sirt3*), Sirtuin 4 (*Sirt4*), and Sirtuin 5 (*Sirt5*) were analysed via quantitative RT-PCR. The results of RT-PCR were normalized to *Actb* gene expression. (B) The total level of AIF and PARP-1 protein was determined using the Western blotting method. Densitometric analysis of the results of Western blot is presented as the mean ± SEM from four independent experiments. Gapdh is presented as a loading control. The typical pictures were presented. *p < 0.05, **p < 0.01, ***p < 0.001, as compared to the control cells, using a one-way ANOVA followed by the Newman-Keuls test.

However, our data demonstrated that an increased level of free radicals activates some other defence mechanisms. It was found that 24 h exposure of PC12 cells to AβO or SKI II resulted in an increase in the expression of genes responsible for anti-oxidative defence, including mitochondria-related SOD2. The effect was more pronounced in the case of SKI II (Fig 4A). Despite these anti-oxidative responses, a significant increase in the level of the apoptosis-inducing factor (AIF), a trigger of the caspase-independent pathway of apoptosis and a protein important in the mitochondrial respiratory chain and metabolic redox reactions, was also found (Fig 4B) without changes in gene expression (S2 Fig). The AIF level remained elevated for up to 96 hours (S3 Fig). We also examined the level of poly(ADP-ribose)polymerase-1 (PARP1), a nuclear protein involved in a number of cellular processes involving mainly DNA repair and programmed cell death. However, there was no change in the PARP-1 protein level (Fig 4B) and its gene expression (S2 Fig) in our experimental conditions. Accumulating evidence suggests the protective effects of sirtuins against common neurological disorders, including Alzheimer’s disease. In our study, the effect of AβO on the expression of genes for mitochondria-related sirtuins (Sirt3, -4 and-5) was analysed. Exposure of cells to AβO for 24 h slightly increased the expression of *Sirt4*; however, 96 h of exposure resulted in an increase in gene expression for all of the examined isoforms (Fig 4C and 4D). Interestingly, the stimulatory effect of SKI II on the expression of *Sirt3*, *-4* and-*5* was observed already after 24 h.

**Fig 5:**
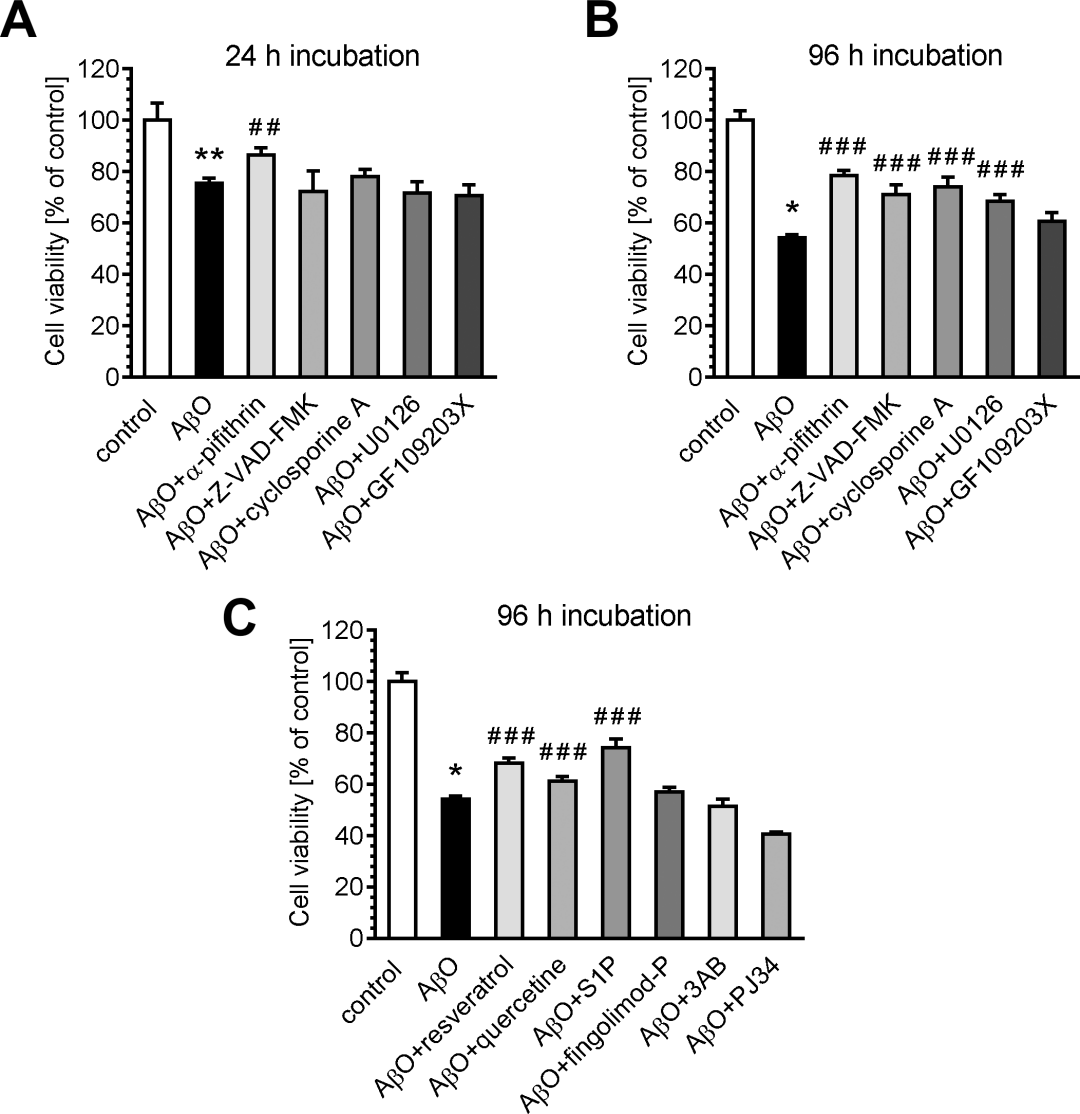
The effect of pharmacologically active compounds on AβO-evoked reduction of PC12 cell viability. PC12 cells were incubated for 24 (A) and 96 h (B, C) in the presence of oligomeric Aβ (AβO, 1 μM) and various pharmacologically active compounds: α-pifithrin (20 μM), Z-VAD-FMK (40 μM), cyclosporine A (2 μM), U0126 (1 μM), GF109203X (1 μM), resveratrol (25 μM), quercetin (100 μM), S1P (1 μM), fingolimod-P (100 pM), 3-aminobenzamide (3AB, 5 mM), PJ34 (20 μM). Cell viability was determined using MTT assay, as described in Methods. *p < 0.05, **p < 0.01, as compared to the control cells, ##*p* < 0.01, ###*p* < 0.001, as compared to AβO-treated group, by using a one-way ANOVA followed by the Newman-Keuls test.

To further elucidate the molecular pathways affected by AβO, the effect of the range of pharmacologically active compounds on AβO-destabilised cell viability was analysed. Our data demonstrated that after 24 h exposure of AβO, only α-pifithrin, an inhibitor of p53, protected cells against death (Fig 5A). However, expression of *Tp53* gene was not changed in these conditions (S4 Fig). After prolonged stress caused by 96 h exposure of AβO, a cytoprotective effect was shown by α-pifithrin as well as by pan-caspase inhibitor Z-VAD-FM, the mitochondrial permeability transition pore blocker (cyclosporine A), the MEK/ERK inhibitor (U0126) and the inhibitor of protein kinase C (GF109203X) (Fig 5B). The protective effect has also been demonstrated by the use of compounds that are both antioxidants and sirtuin activators, i.e. resveratrol and quercetin, both of which attenuated cell death evoked by 96 h exposure to AβO (Fig 5C). Inhibitors of PARP 3-aminobenzamide and PJ34 did not protect against AβO-induced toxicity, thus confirming that PARP is not involved in dead pathway. The product of the SphK-catalysed reaction, sphingosine-1-phosphate (S1P; 1 μM), showed some protective effect after 96 h exposure to AβO, and the modulator of the S1P receptor, fingolimod (FTY-720), had no ameliorating effect in these experimental conditions.

## Discussion

In our study we investigated the relationship between Amyloid beta 1–42 (Aβ_1–42_), sphingosine kinases, mitochondrial sirtuins and other anti-oxidative processes in the molecular mechanism of cell survival and death. Our results demonstrated that the Aβ_1–42_ peptide in the oligomeric form significantly inhibits SphK1 expression and activity, thus leading to disturbances of the sphingolipid biostat and to oxidative stress. Alteration of the balance between sphingosine-1-phosphate (S1P), the product of SphK, and ceramide may be an early key event in cell death. The enhancement of free radical level by Aβ and by inhibition of Sphk may in consequence lead to alteration of Gsk-3β and activation of death signalling. We indicated that pro-survival processes are activated concomitantly, leading to higher gene expression for mitochondrial Sirts (3,4,5) and other anti-oxidative/pro-survival events.

However, all of the demonstrated pro-survival processes are not able to protect the cells against death. An analysis of the anti-oxidative and anti-apoptotic events may be very useful in identifying promising targets for cytoprotection. Moreover, experiments with specific inhibitors indicated that the p53 protein could be responsible for the cell death mechanism at a very early stage of Aβ_1–42_ toxicity (24h). Several studies demonstrated an increase in p53 level in sporadic AD. Upregulated p53 induced, by indirect mechanism, phosphorylation of tau [37]. Furthermore, it was shown that especially Aβ_1–42_ binds to the p53 promoter and influences the expression of several genes [37]. Prolonged time of Aβ_1–42_ action up to 96 h leads to inhibition of SphK1 expression and activity. SphK2 was not altered under the same experimental conditions. SphK1 is mainly a cytosolic enzyme that easily translocates into the plasma membrane during the activation of several growth factor receptors (including IGF, TGF, NGF), and it synthesises S1P which is transported outside the cells and acts as a primary messenger through specific G protein-operated S1P receptors S1P1-S1P7. S1P may also exert an intracellular effect as a secondary messenger. The inhibition of Sphk1 after 96 h of Aβ_1–42_ treatment, which was observed in our study, may lead to the enhancement of free radical production and to lower synthesis of S1P and, in consequence, to disturbances of the S1P-dependent signalling pathways [6, 38].

It was previously demonstrated that Aβ inhibits SphK activity. The treatment of SH-SY5Y cells with Aβ_25–35_ evoked strong inhibition of SphK1 with a concomitant increase in the ceramide level and a decrease in the S1P level. In order to study the involvement of SphK in Aβ-evoked cell death, we incubated PC12 cells with Aβ oligomers and compared the effect with that induced by the SphK inhibitor SKI II. Our data indicated that Aβ reduced cell viability at a similar rate as SKI II.

It was previously postulated that one of the early events in Aβ toxicity is the inhibition of the other most important pro-survival pathway regulated by PI3K/Akt/PKB, followed by a decrease in the phosphorylation of Gsk-3β, thus leading to its higher activity and to hyperphosphorylation of the tau protein [39, 40]. In this study, AD-related Aβ_1–42_ peptides evoked a significant but transient decrease in the total Gsk-3β protein level after 24 h, with no changes after 96 h, and with no significant changes in the level of the (inactive) form phosphorylated on serine 9. The results suggest that during the early period of Aβ_1–42_ toxicity (24 and 96 h), the activity of the PI3K/Akt/Gsk-3β pathway is not significantly altered. However, the transient significant fall of the total protein level of Gsk-3β may have an influence on the metabolic state of cells during the early stage of Aβ toxicity. Our previous data indicated that prolonged exposure of PC12 cells to Aβ (cells transfected with the human gene for APP endogenously liberating a significant pool of Aβ) evoked hyperphosphorylation of MAP tau [41]. Other *in vitro* and *in vivo* experimental models revealed that the relationship between Aβ and Gsk-3β is dependent on time and other experimental conditions [42, 43]. It was demonstrated, for example, that in transgenic mice (PS1×APP) the level of Gsk-3β phosphorylation at Ser9 was significantly increased in young animals (6 months), whereas it was significantly suppressed in aged animals, as compared to wild-type animals [42]. It was described that the acute effect of Aβ on Gsk-3β activity (estimated by Ser9 phosphorylation) in human SH-SY5Y neuroblastoma cells is biphasic, with early activation after 1 h of incubation followed by inactivation after 24 h [44]. In our study, the level of both total Gsk-3β and Gsk-3β phosphorylated on Ser9 was not altered after 96 h of Aβ treatment. A similar effect, as evoked by Aβ peptides, on Gsk-3β protein level was exerted by SphK inhibition.

Aβ_1–42_ and SphK inhibition activated the pro-apoptotic pathway characterized by a significant enhancement of pro-apoptotic *Bax* expression and decrease in gene expression for anti-apoptotic BCL2 proteins. Concurrently, other pro-survival mechanisms were activated. Up-regulation of gene expression for mitochondrial Sirts(3,4,5) was found. Moreover, stimulation of gene expression for mitochondrial superoxide dismutase (Sod2) and catalase was observed as well as enhancement of the protein level of the apoptosis-inducing factor (AIF). AIF is an important mitochondrial protein with anti-oxidative properties. It plays a significant role in integrating respiratory Complex I and III, however, it is also involved in cell death after translocation from the mitochondria into the nucleus [45]. Overactivation of PARP1 was indicated to be responsible for AIF release from mitochondria [46, 47]. However, in our current study PARP1 seems to be involved in DNA repair mechanism. Our data, showing a higher level of AIF with no alteration of PARP1, suggest the involvement of AIF in an adaptive, protective response to the early phase of Aβ toxicity. The functions of mitochondrial proteins are regulated by Sirt3,-4,-5, which are located in these organelles [48]. Sirt3 exhibits NAD^+^-dependent deacetylase activity and deacetylates several proteins containing at least one acetylated lysine. The lack of Sirt3 expression induces several neurological defects, hyperacetylation of mitochondrial proteins and severe metabolic defects [49]. Sirt4 and Sirt5 do not only have NAD^+^-dependent deacetylase activity, but also ADP-ribosylation activity and demalonylation and desuccinylation activity, respectively [22, 29, 50]. Sirts are involved in the regulation of the mitochondrial energy metabolism and probably also mitochondria dynamics and biogenesis [29]. Sirt3 exerted an anti-oxidative effect by direct interaction or deacetylation of the mitochondrial enzyme isocitrate dehydrogenase 2 (IDH2) and by regulating the expression and activity of SOD2. Sirt3 is involved in an important anti-oxidative pathway connected with glutathione reductase (GR)/glutathione peroxidase (GPX). The higher expression of Sirt3,-4,-5 that we observed in our study suggests the activation of important anti-oxidative processes.

Despite the activation of Sirt3,-4,-5 and other mitochondrial pro-survival events, a significant population of cells died. Experiments with specific inhibitors demonstrated that protein p53 is responsible for cell death at a very early stage of toxicity of Aβ_1–42_, and that the inhibition of SphK1 and lower S1P synthesis can be another important molecular event. Previous data indicated that p53 is significantly engaged in cell death signalling by regulating genes for Bax and Gadd45 [51, 52]. In this study, the inhibitor of p53 (α-pifithrin) significantly enhanced viability of cells subjected to Aβ_1–42_. P53 is a pleiotropic protein involved in cell differentiation and apoptosis. It is generally accepted that p53 plays an important role in neuronal death in neurodegenerative disorders [53, 54]. This protein exerted several properties with regard to mitochondrial and nuclear processes, including involvement in the DNA repair mechanism [53, 55]. However, p53-overexpressing cells showed reduced mitochondrial function, including a reduction in reserve and maximal respiratory capacity. A truncated mitochondria-localising p53 was generated, and its conformational alterations influenced cell function [56].

Moreover, our study demonstrated that the inhibition of SphK1 and lower S1P synthesis are important and early events in Aβ_1–42_-evoked cell death. Inhibition of ShpK1 kinase is also responsible for the activation of oxidative stress and for neuronal cell death in an experimental model of Parkinson’s disease [36, 57]. It was previously suggested that loss of neuroprotective S1P and inhibition of SphK activity are crucial events in AD pathogenesis [6, 11]. The study by Couttas et al. [6] indicated that the S1P/sphingosine ratio was 66% and 64% lower in Braak stage III/IV in the hippocampus, and that SphK1 and SphK2 activity was significantly reduced in the hippocampus. However, Takasugi et al. [12] showed that SphK2 activity is enhanced by fibrillary Aβ_1–42_ and in the AD brain. The S1P pool synthesised by SphK2 has been suggested as being responsible for BACE 1 activation and Aβ_1–42_ liberation. Our data showed that oligomeric Aβ_1–42_ evokes exclusively SphK1 inhibition. Up until now, the role of SphK1, SphK2 and the involvement of S1P in neurodegeneration and neuroprotection have not been fully elucidated. In our study, S1P exerted a neuroprotective effect at 1 μM. Recently, van Echten-Deckert et al. [58] described the negative and positive action of S1P in the brain. Our results demonstrated that agonist of S1P receptors, fingolimod (FTY720), has no effect on Aβ_1–42_ toxicity. However, its chronic administration prevents cognitive impairment in the rat model of AD [59]. On the basis of our results, we suggest that the inhibition of SphK1 by Aβ_1–42_ is a crucial point in activating the molecular mechanisms of cell death. An analysis of the selected inhibitors and potential protectants indicates that the activation of protein kinase C (PKC) and extracellular signal-regulated kinase (ERK), then alteration of mitochondrial membrane permeability, participate in caspase-dependent cell death evoked by Aβ_1–42_. Kim and Choi (2010) described the pathological roles of serine/threonine kinases in human disease [60]. It is known that ERKs, as the other members of MAPK family, phosphorylate various transcription factors, including p53, ELK1, c-Jun, as also cytoskeleton proteins, and play an important role in neurodegenerative diseases. The activation of neuronal ERK in AD links oxidative stress to abnormal phosphorylation [61]. Protein kinase C was suggested as an early biochemical marker in AD [62]. Kim et al. (2011) published data indicating that PKC promotes production of secretory form of APP and regulates Aβ production and its clearance [63]. In our study, we have used the non-selective PKC inhibitor GF109203X which protects cells against Aβ toxicity. This inhibitor reduces the degradation of IKBα and mitochondrial cytochrome c release, and this effect is highly correlated with changes in PKCδ and PKCε [64]. However, the role of particular PKC isoforms in Aβ toxicity is not fully elucidated. In addition, our data indicated that resveratrol and quercetin enhance significantly cell survival under Aβ_1–42_ toxicity. Previously published data demonstrated that resveratrol exhibits neuroprotective benefits in animal model of AD. Resveratrol promoted non-amyloidogenic cleavage of APP by α-secretase-, enhanced clearance of Aβ, and reduced neuronal damage [65].

Summarizing, on the basis of our data, it is possible to suggest that the p53 inhibitor and activator of specific S1P receptor(s) and Sirts should be considered for cytoprotection and, in consequence, for the improvement of the AD therapeutic strategy.

## Supporting Information

S1 Fig The effect of prolonged incubation in the presence of oligomeric Aβ on level and phosphorylation of Gsk-3β. PC12 cells were incubated in the presence of oligomeric Aβ (AβO, 1 μM) for 96 h. The total level of Gsk-3β protein and phosphorylation at Ser9 were determined using the Western blotting method. Densitometric data were normalized to total protein level, as determined by Ponceau S staining. Data represent the mean value ± S.E.M. for 4–8 independent experiments. The typical pictures were shown. Gapdh is presented as a loading control.
(TIF)

S2 Fig The effect of oligomeric Aβ on expression of genes for AIF and PARP1. PC12 cells were incubated in the presence of oligomeric Aβ (AβO, 1 μM) for 24 h. The levels of mRNA for AIF and PARP-1 were analysed via quantitative RT-PCR. The results of RT-PCR were normalized to *Actb* gene expression. Data represent the mean value ± S.E.M. for 3–4 independent experiments.
(TIF)

S3 Fig The effect of prolonged incubation in the presence of oligomeric Aβ on level of AIF. PC12 cells were incubated in the presence of oligomeric Aβ (AβO, 1 μM) for 96 h. The total level of AIF protein was determined using the Western blotting method. Densitometric data were normalized to total protein level, as determined by Ponceau S staining. Data represent the mean value ± S.E.M. for three independent experiments. The typical pictures were shown. Gapdh is presented as a loading control. *p < 0.05, as compared to the control cells, using Student t test.
(TIF)

S4 Fig The effect of oligomeric Aβ on expression of gene for Tp53. PC12 cells were incubated in the presence of oligomeric Aβ (AβO, 1 μM) for 24 and 96 h. The level of mRNA of *Tp53* was analysed via quantitative RT-PCR. The results of RT-PCR were normalized to *Actb* gene expression.
(TIF)
