# Supplementary figures and images for: The Molecular Mechanism of Amyloid β42 Peptide Toxicity: The Role of Sphingosine Kinase-1 and Mitochondrial Sirtuins
Source: PLoS One. 2015 Sep 3;10(9):e0137193. doi: 10.1371/journal.pone.0137193 (PMC4567180; doi:10.1371/journal.pone.0137193)

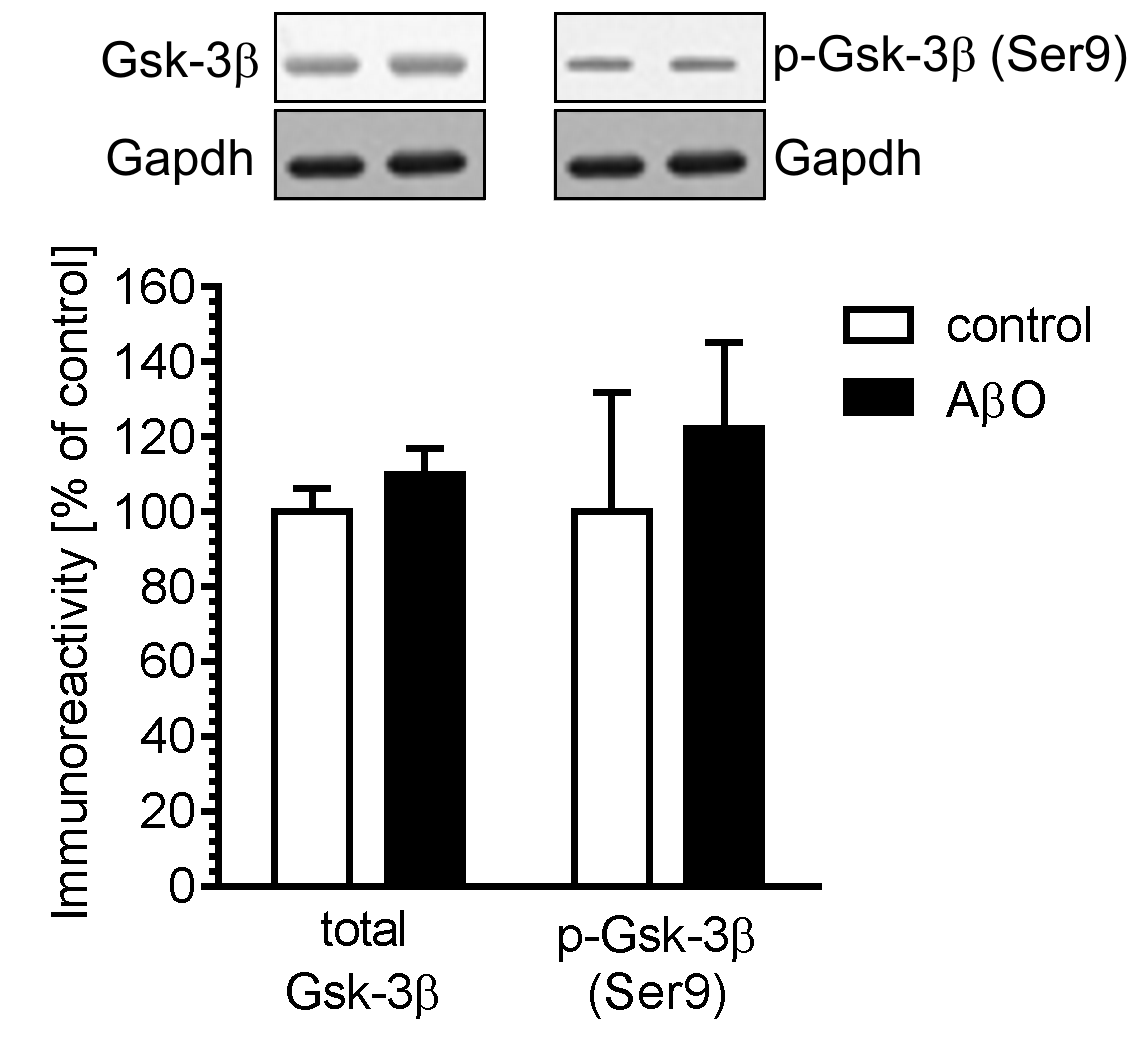

Supplement: S1 Fig — PC12 cells were incubated in the presence of oligomeric Aβ (AβO, 1 μM) for 96 h. The total level of Gsk-3β protein and phosphorylation at Ser9 were determined using the Western blotting method. Densitometric data were normalized to total protein level, as determined by Ponceau S staining. Data represent the mean value ± S.E.M. for 4–8 independent experiments. The typical pictures were shown. Gapdh is presented as a loading control. (TIF) [file pone.0137193.s001.tif]

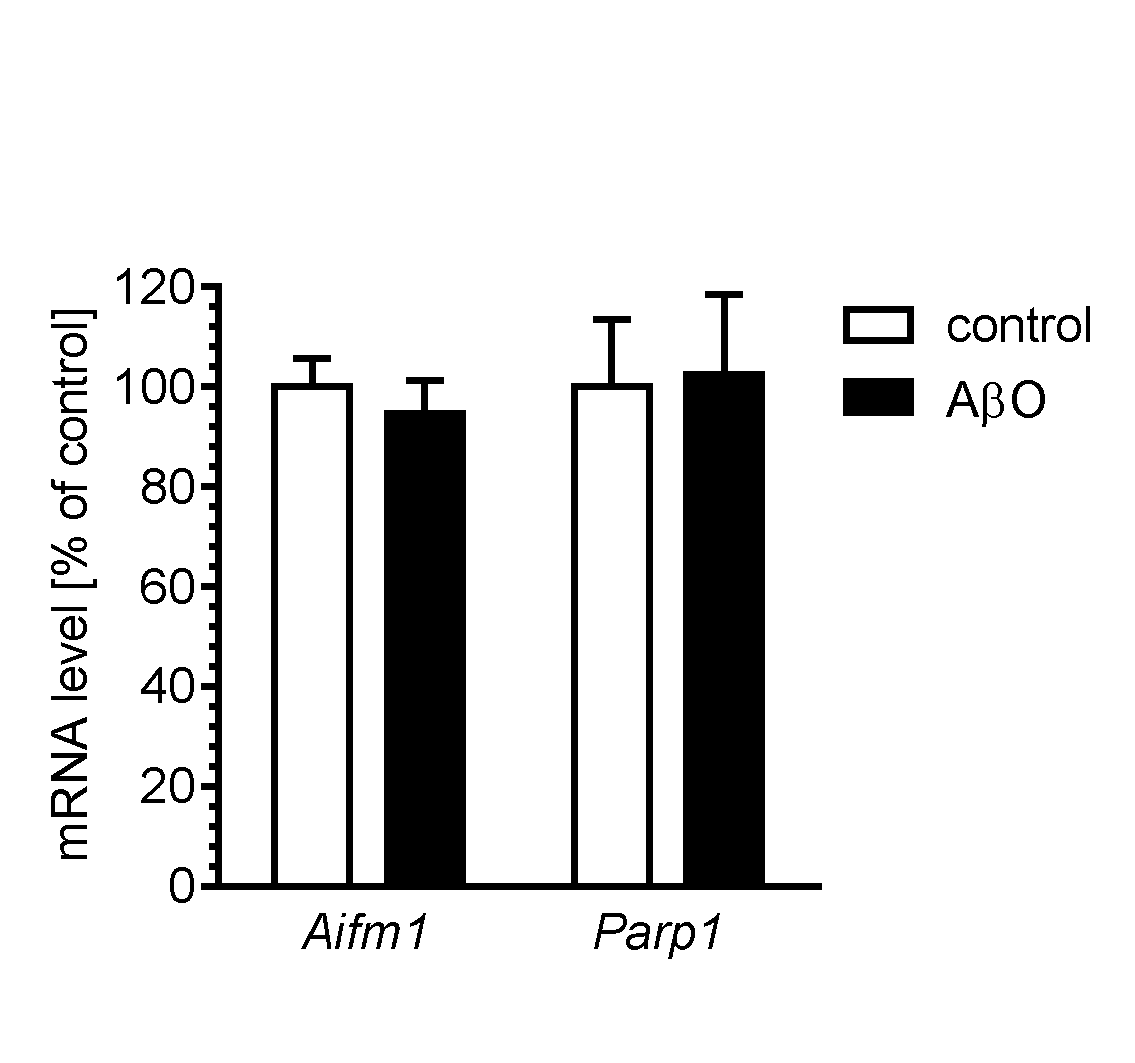

Supplement: S2 Fig — PC12 cells were incubated in the presence of oligomeric Aβ (AβO, 1 μM) for 24 h. The levels of mRNA for AIF and PARP-1 were analysed via quantitative RT-PCR. The results of RT-PCR were normalized to Actb gene expression. Data represent the mean value ± S.E.M. for 3–4 independent experiments. (TIF) [file pone.0137193.s002.tif]

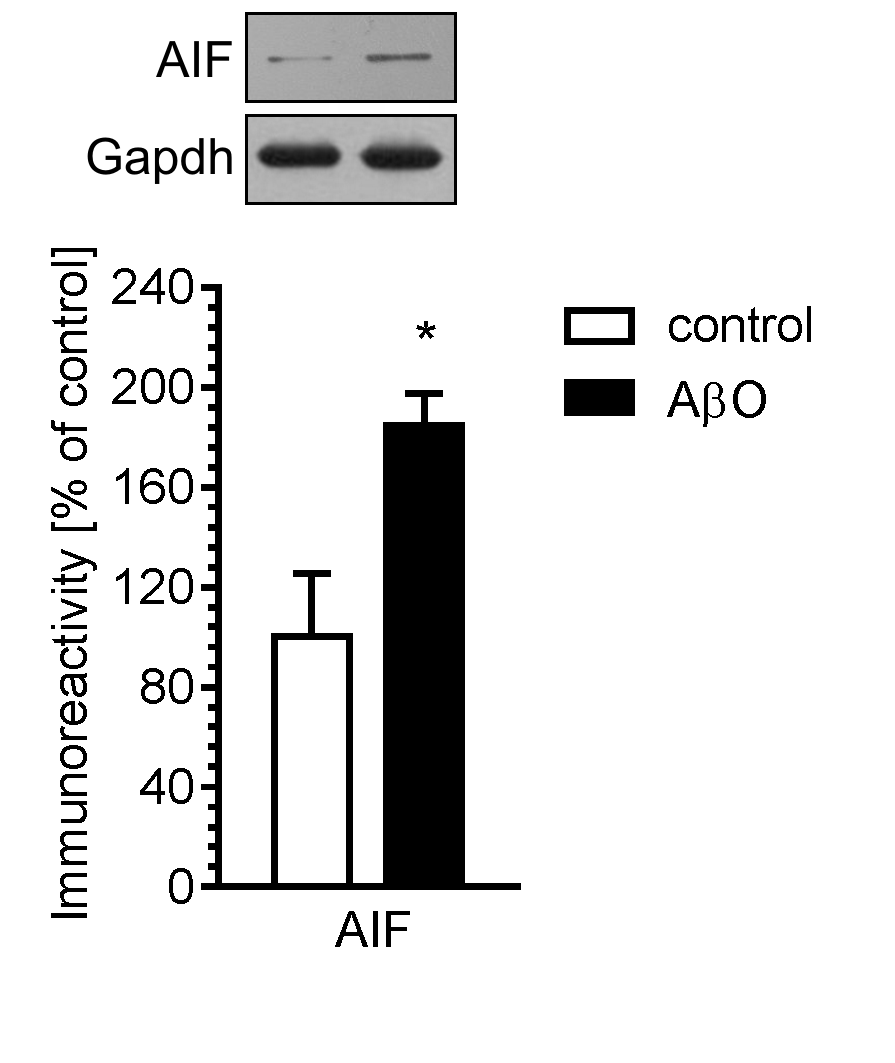

Supplement: S3 Fig — PC12 cells were incubated in the presence of oligomeric Aβ (AβO, 1 μM) for 96 h. The total level of AIF protein was determined using the Western blotting method. Densitometric data were normalized to total protein level, as determined by Ponceau S staining. Data represent the mean value ± S.E.M. for three independent experiments. The typical pictures were shown. Gapdh is presented as a loading control. *p < 0.05, as compared to the control cells, using Student t test. (TIF) [file pone.0137193.s003.tif]

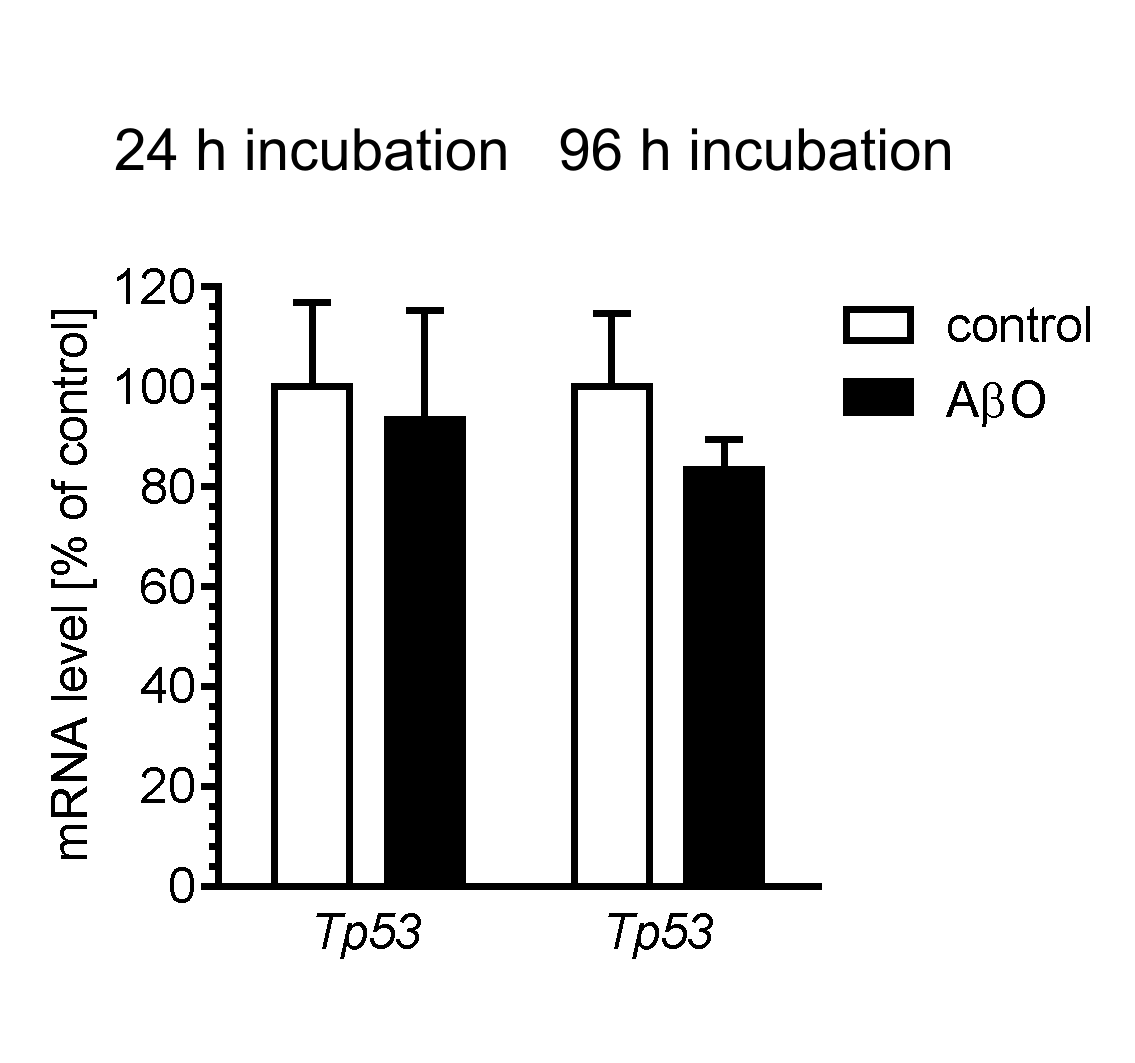

Supplement: S4 Fig — PC12 cells were incubated in the presence of oligomeric Aβ (AβO, 1 μM) for 24 and 96 h. The level of mRNA of Tp53 was analysed via quantitative RT-PCR. The results of RT-PCR were normalized to Actb gene expression. (TIF) [file pone.0137193.s004.tif]
